# Internet use, socioeconomic status, and smoking behaviors among Chinese men: A secondary longitudinal dataset analysis of the China Family Panel Study

**DOI:** 10.18332/tid/225231

**Published:** 2026-07-31

**Authors:** Hao Yuan, Fanrong Wu

**Affiliations:** 1 School of Sociology and Political Science, Shanghai University, Shanghai, China

**Keywords:** smoking, socioeconomic status, internet use, moderating effect

## Abstract

**INTRODUCTION:**

Despite intensified tobacco-control efforts, male smoking prevalence in China has decreased, but remains high at 45%. With the rapid development of the Internet, the influence and strength of Internet use on smoking behaviors are unclear in China. This study tests whether socioeconomic status and Internet use, respectively, are positively associated with smoking behaviors among Chinese men, and examines the moderating effects of Internet use in the correlation between socioeconomic status and smoking behaviors.

**METHODS:**

This is a secondary dataset analysis using data from the China Family Panel Study (2014–2022), involving 8916 male participants aged ≥16 years. Key variables included daily cigarette consumption (outcome), socioeconomic status (household income, education level, occupation), and Internet use (access, online chatting/learning/shopping/entertainment). This study applies two-way fixed-effects negative-binomial models to assess the moderating effect of Internet use.

**RESULTS:**

Higher SES (higher income: IRR=1.03; 95% CI: 1.02–1.04, p<0.001; education level: IRR=1.01; 95% CI: 1.01–1.02, p<0.001) is associated with increased daily cigarette consumption. Internet use is also positively related to daily cigarette consumption (IRR=1.03, 95% CI: 1.02–1.04, p<0.001). Income strengthens the association between Internet use and smoking behaviors (interaction effect: IRR=1.005; 95% CI: 1.001–1.009, p<0.01), while more years of schooling weakens this relationship (interaction effect: IRR=0.998; 95% CI: 0.996–0.999, p<0.001). Moreover, being out of the labor force can also weaken the Internet-smoking link (interaction effect: IRR=0.962; 95% CI: 0.947–0.977, p<0.001).

**CONCLUSIONS:**

Socioeconomic status and Internet use are positively related to smoking behaviors. Economic advancement can strengthen the impact of Internet use on DCC, but education level and employment status make Internet use much less important for smoking behaviors. Therefore, tobacco control interventions could incorporate online outreach approaches, with targeted initiatives developed specifically for males of high socioeconomic status with frequent online access. Policymakers are strongly encouraged to develop and implement more comprehensive, forward-looking tobacco control strategies that are specifically tailored to the online environment.

## INTRODUCTION

Smoking has been recognized as an urgent threat to human health with millions of people dying annually from smoking-related diseases, and increased risk of increased susceptibility to cardiovascular diseases, adverse effects on reproductive and developmental health, and a potential link to diabetes and other conditions^1^. In recent years, the Chinese government has implemented a series of nationwide tobacco-control measures, establishing a multi-level tobacco-control framework^2^. However, the smoking rate among Chinese males is still about 50.5%, with a significant gender disparity with males the primary smokers^3^. Smoking is deeply ingrained in male social practices, regarded as a symbol of masculinity and assisting interpersonal interactions^4^. This is partly attributable to the cultural importance of smoking in Chinese social settings, where cigarette gifting significantly contributes to the high smoking prevalence and perceived social benefits often outweigh health risks, thereby fostering a socially accepted smoking behavior^5^.

Socioeconomic status (SES) is widely acknowledged as a structural determinant of smoking behavior. In high-income countries, smoking prevalence and intensity are inversely associated with SES. Research indicates that individuals with lower SES exhibit higher rates of smoking and face an elevated risk of mortality, even when smoking at low intensity. Individuals with low SES are more inclined to adopt unhealthy habits such as smoking, drinking, insufficient physical activity, and unhealthy eating patterns^6^. Conversely, high-SES groups typically exhibit healthier behaviors^7^.

However, studies in developing countries reveal a positive association between SES and tobacco use^8,9^. Studies have shown that individuals from lower income households exhibit a higher likelihood of smoking, which can subsequently lead to increased poverty and health costs^8^. Research in China indicates that individuals with lower socioeconomic status (SES) are more likely to engage in unhealthy lifestyles, which in turn is associated with a higher risk of death and cardiovascular diseases. Individuals with lower SES exhibit a lower probability of engaging in health-risk behaviors, whereas higher-SES groups are more prone to smoking^9^.

Why does cigarette smoking spread so differently through these two kinds of societies over time? It can be attributed to the fact that China and other high-income countries are at different stages of tobacco diffusion^10^. China is still at the early stages, where smoking is still regarded as a symbol of status and masculinity^4^. In high-income countries, higher socioeconomic groups begin to quit smoking following health awareness and anti-smoking campaigns, leading to a decline in male smoking prevalence. But smoking prevalence remains persistently high among lower socioeconomic groups, creating widening health disparities based on socioeconomic status^11^.

Over the past decade, Internet penetration in China has significantly increased, with the number of internet users growing from 564 million in 2012 to over 1.079 billion by June 2023, and the internet penetration rate soaring from 42.1% to 76.4%^12^. Internet use typically includes online chatting, shopping, entertainment, and gaming. Meta-analysis evidence demonstrates that increased exposure to tobacco advertising and promotional content on the Internet elevates the likelihood of smoking initiation, since social media, virtual communities, online games, and streaming platforms all offer opportunities for social learning of smoking^13,14^. Digital communities may facilitate the spread of cigarette use^15^. And spending more time on social media is significantly associated with more smoking behaviors among young people^16^.

A robust positive SES gradient in Internet use has been well documented^17^. Advanced digital skills, such as using the Internet for news, job searching, product information acquisition, and work-related activities, are strongly related to education level and household income^18^. High-SES individuals typically possess superior digital literacy, thus enabling them to actively search for, evaluate, and subscribe to smoking-cessation or health-promotion content, thereby reducing their exposure to pro-tobacco messages^19^. In contrast, low-SES individuals tend to engage more in online games and entertainment, which may contribute to worse Internet addiction and more cigarette consumption^20^.

In addition, high-income groups are also more likely to obtain online health information, conferring additional advantages in health communication and tobacco control^21,22^. High-SES groups are in a better position to offer social support, exert normative pressure against addictive behaviors, and moderate the effect of Internet use on healthy behaviors^23^. Adolescents from low-SES families are more likely to exhibit problematic internet use, as evidenced by a significant positive link between low SES and excessive social media use. However, this link does not exist among adolescents from high-SES backgrounds, suggesting that higher SES may act as a protective factor against addiction risk^24^. Thus, SES appears to exert a systematic weakening effect on Internet-induced addictive behaviors.

Thus, this study aims to systematically investigate whether SES and Internet use are, respectively, associated with daily cigarette consumption (DCC) among adult men in China. Specifically, we aim to assess whether higher individual SES – typically measured by a combination of education level, occupational category, and household income – is associated with an increase in daily cigarette consumption. Concurrently, it will examine whether the frequency of individual Internet use is positively correlated with DCC. Furthermore, this study will analyze the potential moderating role of SES in the relationship between Internet use and smoking behavior. That is, it will assess whether SES can enhance, attenuate, or alter the strength of the impact of Internet use on Chinese men’s DCC.

## METHODS

### Data source

This secondary dataset analysis utilizes the dataset from the China Family Panel Study (CFPS), a longitudinal project overseen by the Institute of Social Science Survey at Peking University. CFPS is designed to monitor societal, economic, demographic, educational, and health changes in China by collecting data at the individual, household, and community levels. Initiated in 2010, the study conducts follow-up surveys biannually, encompassing a sample size of 16000 households across 25 provinces, municipalities, and autonomous regions in China. Considering the comprehensive data on internet usage available for the years 2014, 2016, 2018, 2020, and 2022, our analysis is confined to these five waves. The resulting unbalanced panel dataset includes 68565 male respondents aged ≥16 years.

### Measurements

*Dependent variable*

The dependent variable is DCC, derived from the CFPS question: ‘During the past month, on average, how many cigarettes did you smoke per day?’. Non-smokers are coded 0. Approximately 50% of male respondents report being non-smokers. Among smokers, 20% of them smoke more than 20 cigarettes per day, and fewer than 0.5% smoke more than 40 cigarettes per day. Smoking more than 20 cigarettes per day (1 pack) or 40 cigarettes per day is considered a clinically meaningful threshold for heavy smoking^25^.

*Independent variables*

The key explanatory variables are Internet use and SES. Internet use is operationalized as the sum of five indicators: Internet access (0=no, 1=yes); online chatting (0=no, 1=yes); frequency of online-learning (0=never, 1=sometimes, 2=daily); frequency of online-entertainment (0=never, 1=sometimes, 2=daily); frequency of online-shopping (0=never, 1=sometimes, 2=daily); with higher values denoting more intensive internet engagement.

SES is operationalized through three conventional indicators: household income (log), occupational position, and years of schooling based on the education level attained.

*Covariates*

To mitigate omitted-variable bias, we controlled for age, urban residence, province-level region, life-satisfaction score, marital status, presence of chronic disease, and employment status.

### Statistical analysis

The dependent variable, namely DCC, is a count outcome that exhibits over-dispersion and an excess of zeros. Consequently, two-way fixed-effects negative-binomial panel regressions are estimated to assess the effects of SES and Internet use on smoking behavior. All statistical analyses are conducted using Stata statistical software (Version 17). Statistical significance is set at p<0.05 in each analysis.

Cross-sectional analyses fail to account for unobserved, time-invariant individual heterogeneity or omitted variables that vary across waves yet are common to all individuals. These factors may simultaneously affect both the independent and dependent variables, leading to biased coefficient estimates. Owing to the count nature of the dependent variable and its excess zeros, a two-way fixed-effects negative-binomial panel model is employed. Since this specification accounts for within-individual variation in both the dependent and independent variables, observations with no temporal change in cigarette consumption are excluded, which leads to a reduced effective sample size.

## RESULTS

Table 1 illustrates the temporal trend in male smoking behavior. DCC decreased markedly, from 8.8 cigarettes in 2014 to approximately 8.2 in 2016 and 2018, and subsequently dropped sharply to 7.3 in 2020 and 6.9 in 2022. Over the nine-year period, average consumption decreased by approximately two cigarettes, indicating a 21.7% reduction.

**Table 1:** Changes in smoking behaviors, Internet use and socioeconomic status between 2014 and 2022, CFPS (N=68565)

*Variable*	*2014*	*2016*	*2018*	*2020*	*2022*
DCC	8.76	8.18	8.27	7.29	6.86
Log of household income	8.68	9.45	9.71	9.88	10.03
Years of schooling	8.22	8.53	8.75	8.45	8.47
Internet use	1.41	2.57	2.66	2.90	3.56

DCC: daily cigarette consumption. Household income: annual disposable income of the respondent’s household. Years of schooling: the total number of years of formal education completed by the respondent. Internet use: the sum of Internet access (0=no, 1=yes), online chatting (0=no, 1=yes), frequency of online-learning (0=never, 1=sometimes, 2=daily), frequency of online-entertainment (0=never, 1=sometimes, 2=daily), frequency of online-shopping (0=never, 1=sometimes, 2=daily).

Log of household income exhibited a steady increase from 8.7 in 2014 to 10 in 2022. Years of schooling rose from 8.2 years in 2014 to 8.7 years in 2018, experienced a slight decline to 8.5 years in 2020 and 2022. Internet use, a sum of five indicators, rose sharply from 1.4 in 2014 to 3.6 in 2022, reflecting the sustained expansion of broadband infrastructure and the widespread adoption of digital devices (more details can be found in the Supplementary file).

Table 2 reports the results from the fixed-effects negative-binomial regression models. First, household income displays a similarly stable, positive association with DCC in Model 1 (Baseline model: DCC, all SES indicators, internet use, and full control variables). One-unit rise in log of household income raises DCC by about 3.1% (incidence rate ratio, IRR=1.031; 95% CI: 1.023–1.040, p<0.001). Increases in the year of schooling result in higher DCC (IRR=1.013; 9 5 % CI: 1.009–1.017, p<0.001). Occupational type also matters: relative to workers, farmers (IRR=0.873; 95% CI: 0.844–0.902, p<0.001) smoke significantly less. The non-employed males have the lowest levels of DCC (IRR=0.587; 95% CI: 0.562–0.615, p<0.001), underscoring the workplace as a key smoking-promoting environment. Internet use is also significantly associated with DCC (IRR=1.034; 95% CI: 1.027–1.040, p<0.001).

**Table 2:** Negative-binomial regression analyses of the correlations of SES and Internet use with DCC among Chinese adult men, CFPS, 2014–2022

*Variables*	*Model 1 Two-way fixed-effects model on DCC*		*Model 2 Moderating effect of income on DCC*		*Model 3 Moderating effect of education on DCC*		*Model 4 Moderating effect of employment on DCC*		*Model 5 Moderating effect of SES on DCC*	
	*IRR*	*95% CI*	*IRR*	*95% CI*	*IRR*	*95% CI*	*IRR*	*95% CI*	*IRR*	*95 % CI*
**Household income**	1.031***	1.023–1.040	1.036***	1.028–1.044	1.030***	1.022–1.039	1.031***	1.023–1.040	1.036***	1.028–1.044
**Year of schooling**	1.013***	1.009–1.017	1.012***	1.008–1.016	1.014***	1.010–1.018	1.013***	1.009–1.017	1.014***	1.010–1.018
**Occupation type**										
Working class (ref.)	1		1		1		1		1	
Manager	1.024	0.981–1.069	1.022	0.979–1.067	1.026	0.983–1.072	1.021	0.978–1.066	1.021	0.978–1.066
Professional	0.986	0.946–1.028	0.985	0.945–1.027	0.988	0.948–1.030	0.981	0.942–1.022	0.982	0.943–1.023
Farmer	0.873***	0.844–0.902	0.872***	0.843–0.902	0.875***	0.847–0.905	0.882***	0.853–0.911	0.883***	0.854–0.913
Non-employed	0.587***	0.562–0.615	0.589***	0.563–0.616	0.589***	0.563–0.616	0.579***	0.553–0.607	0.582***	0.555–0.610
**Internet use**	1.034***	1.027–1.040	1.033***	1.026–1.039	1.057***	1.044–1.069	1.037***	1.031–1.043	1.060***	1.047–1.072
**Internet use × household income**			1.005**	1.001–1.009					1.005***	1.001–1.009
**Internet use × year of schooling**					0.998***	0.996–0.999			0.997***	0.995–0.999
**Internet use × non-employed**							0.962***	0.947–0.977	0.964***	0.949–0.979
**Total**, n	41164		41164		40846		40846		40846	

IRR: incidence rate ratio. DCC: daily cigarette consumption. SES: socioeconomic status. All models adjust for age, gender, marital status, urban/rural residence, chronic disease status, life satisfaction, survey year, and geographical region. In all the interaction effect models, independent variables are centered with their means.

* p<0.05.

** p<0.01.

*** p<0.001.

To assess how social structure conditions the effect of Internet use on smoking, Model 2 (Main effects + household income × Internet use interaction) introduces an interaction term between household income and Internet use. It shows that household income amplifies the Internet–smoking correlation (IRR=1.005; 95% CI: 1.001–1.009, p<0.01); wealthier men experience a larger increase in daily cigarette consumption for a given increment in Internet use. Model 3 (Main effects + years of schooling × Internet use interaction) further includes an interaction term between years of schooling and Internet use. Each additional year of education attenuates the positive correlation between Internet use and DCC by 0.2% (IRR=0.998; 95% CI: 0.996–0.999, p<0.001); the smoking-inducing effect of Internet use is thus weaker among highly educated men in China. Model 4 (Main effects + non-employment status × Internet use interaction) tests the interaction effect of employment status on the Internet-smoking relationship. It shows that non-employed men smoke fewer cigarettes than the employed for a given increment in Internet use (IRR=0.962; 95% CI: 0.947–0.977, p<0.001). Model 5 (Full model: all main effects + three SES–Internet interaction terms) includes these three interaction effects on DCC. The results are similar to those in the previous three models, indicating that the interaction effects of SES with Internet use are relatively robust.

## DISCUSSION

In light of China’s ongoing lifestyle transformation and the entrenched alcohol- and tobacco-friendly culture, this study aims to explore how SES and Internet use interact to shape the daily cigarette consumption of Chinese men, using a secondary individual-level panel dataset from the China Family Panel Study (2014–2022).

This study reveals that household income exhibits an ‘anti-gradient’ pattern, also observed in developing countries^8,9^. Rising household income and higher level of education are both associated with increased cigarette consumption. While schools emphasize the harms of tobacco and maintain a relatively low smoking prevalence, the transition from student to employee exposes Chinese men to new social settings where cigarettes often function as social currency, thereby increasing their likelihood of smoking.

We noted that individuals out of the labor market smoke significantly fewer cigarettes than their employed counterparts, highlighting the workplace as a crucial context that promotes smoking^26^. The occupational gradient is primarily driven by the disparity between farmers and non-agricultural workers; within the non-agricultural sector, differences across occupational strata are negligible. Moreover, education weakens the association between Internet use and smoking. Each additional year of schooling reduces the marginal effect of Internet use on cigarette consumption. Highly educated men tend to apply the Internet for self-improvement and selectively screen out pro-tobacco content. Further analysis indicates that individuals with a lower level of education are more vulnerable to detrimental online social interactions^27^. Therefore, digital health initiatives and clinician-patient online interaction programs should place greater emphasis on the subgroup with lower level of education.

Our study also noted that household income amplifies the growth in cigarette consumption driven by Internet access. As the income of male consumers rises, their online activities, including online social interaction and digital entertainment, lead to greater cigarette consumption. Consequently, individuals with low income who do not utilize the Internet, demonstrate the lowest level of cigarette consumption. These findings are inconsistent with prior research that reported the protective function of SES in addiction risk^24^. Therefore, Internet-based smoking cessation interventions with precise targeting and advanced digital smoking cessation tools should prioritize male internet users from higher socioeconomic backgrounds^28^.

We noted that the effect of internet use on smoking behavior is significantly weaker among the non-employed population. The majority of this group consists of students, unemployed individuals, and retirees, who typically have a more limited scope of online activities. As a result, they are exposed to fewer tobacco advertisements and pro-smoking stimuli, which reduces the strength of the association between Internet use and cigarette consumption. Policymakers should prioritize safeguarding the newly employed individuals from tobacco-related content, to maintain their relatively low risk of smoking.

### Limitations

Several limitations warrant particular attention. First, smoking behavior was measured solely by daily cigarette consumption, overlooking the price of cigarettes and the symbolic significance of tobacco, which influences smoking behaviors among Chinese men. Second, limitations in data and questionnaire design hindered an analysis of the impact of interactions within virtual or gaming communities on smoking, despite the increasing prominence of these interactions in online settings. Third, rapid advances in Internet-based cessation tools and digital interventions have yet to be integrated into longitudinal analyses. Future research should incorporate these evolving technologies to refine our understanding of online determinants of tobacco use.

## CONCLUSIONS

This study elucidates the complex interplay between SES, Internet use, and cigarette consumption among Chinese men. It reveals an ‘anti-gradient’ pattern where higher household income and education level correlate with increased smoking, largely driven by workplace socialization and the role of cigarettes as social currency. Crucially, it demonstrates that SES significantly moderates the impact of digital engagement on smoking habits. Education serves as a protective buffer, enabling individuals to filter out pro-tobacco content, whereas higher income exacerbates the smoking-inducing effects of online entertainment and social interaction. Conversely, non-employed individuals exhibit lower consumption due to restricted online exposure.

## Supplementary Material



## Data Availability

The data supporting this research are available from the following sources: The China Family Panel Studies (CFPS) data are publicly available to registered researchers. Datasets can be downloaded free of charge from the Peking University Open Research Data Platform (doi:10.18170/DVN/45LCSC) or the official CFPS portal https://cfpsdata.pku.edu.cn.
