# Supplementary material for: Internet use, socioeconomic status, and smoking behaviors among Chinese men: A secondary longitudinal dataset analysis of the China Family Panel Study
Source: Tob Induc Dis. 2026 Jul 31;24:10.18332/tid/225231. doi: 10.18332/tid/225231 (PMC13426431; doi:10.18332/tid/225231)
Supplement: Supplementary file 1 [file TID-24-130-s1.pdf]

Tobacco Induced Diseases

Supplementary file

© 2026 Yuan H. and Wu F.

DOI:

10.18332/tid/225231

The content has been provided by the author(s) and has not been reviewed, verified, or endorsed by European Publishing. It may not have undergone peer review. The views, opinions, and recommendations expressed are solely those of the author(s) and do not necessarily reflect the position of European Publishing. European Publishing accepts no responsibility or liability for any consequences arising from the use of, or reliance on, this content.

Table 1. ***Definitions, Coding and descriptive statistics of the Variables of the secondary dataset of the China Family Panel Study, 2014-2022 (N=68565)***

Variable Name	Variable type	Coding and Full Definition	Response Categories / Range & Unit	Mean±S.D. for Overall Population
Daily Cigarette Consumption	Count Variable	Number of cigarettes smoked by the respondent per day	Range: Positive integers ≥ 0, Unit: Cigarettes	7.9 ± 10.3
Occupational Status	Categorical Variable	Classification of the respondent's current occupational status	Non-employ, Working Class,Manager,Professional/Technical Worker,Farmer, Non-employed	-
Household Income	Continuous Variable	Annual disposable income of the respondent's household	Range: ≥ 0, Unit: 10,000 Yuan/year	9.6 ± 2.0
Years of Schooling	Count Variable	Total years of formal schooling received by the respondent	Range: Positive integers ≥ 0, Unit: Years	8.0 ± 4.6
Internet Access	Binary Variable	Whether the respondent has access to usable internet	All response categories: 0 = No, 1 = Yes	0.5 ± 0.5
Online Chatting	Binary Variable	Whether the respondent uses social networking service platforms	All response categories: 0 = No, 1 = Yes	0.4 ± 0.5
Online Learning	Ordinal Categorical Variable	Frequency of the respondent's online learning via the internet	All response categories: 0 = Never, 1 = Occasionally, 2 = Daily	0.3 ± 0.6
Online Shopping	Ordinal Categorical Variable	Frequency of the respondent's online shopping via the internet	All response categories: 0 = Never, 1 = Occasionally, 2 = Daily	0.3 ± 0.5
Online Entertainment	Ordinal Categorical Variable	Frequency of the respondent's online entertainment via the internet	All response categories: 0 = Never, 1 = Occasionally, 2 = Daily	0.5 ± 0.7
Composite Internet Usage Score	Continuous Variable	Sum of the scores of the above 5 internet usage indicators, used to measure the respondent's overall internet usage level	Range: 0-8 , Unit: Composite Score	2.0 ± 2.3
Age	Continuous Variable	Age of the respondent in full years	Range: ≥ 18, Unit: Years	48.4 ± 15.8
Employment Status	Binary Variable	Whether the respondent is currently employed	All response categories: 0 = Unemployed, 1 = Employed	0.9 ± 0.4
Urban Area	Binary Variable	Type of area the respondent currently resides in	All response categories: 0 = Rural Area, 1 = Urban Area	0.5 ± 0.5
Marital Status	Binary Variable	The respondent's current marital status	All response categories: 0 = Unmarried/Cohabiting/Divorced/Widowed, 1 = Married	0.8 ± 0.4
Party Membership	Binary Variable	Whether the respondent is a member of the Communist Party of China	All response categories: 0 = Non-member, 1 = Member	0.1 ± 0.4
Chronic Disease Status	Binary Variable	Whether the respondent has been diagnosed with a chronic disease	All response categories: 0 = No Chronic Disease, 1 = Has Chronic Disease	0.1 ± 0.4
